# Relationship between patient activation measurement and self-rated health in patients with chronic diseases

**DOI:** 10.1186/s12875-020-01301-y

**Published:** 2020-11-04

**Authors:** Nina Tusa, Hannu Kautiainen, Pia Elfving, Sanna Sinikallio, Pekka Mäntyselkä

**Affiliations:** 1 Institute of Public Health and Clinical Nutrition, University of Eastern Finland, P.O. Box 1627, FI-70211 Kuopio, Finland; 2 Primary Health Care Unit, Kuopio University Hospital, Kuopio, Finland; 3 Siilinjärvi Health Center, Siilinjärvi, Finland; 4 Folkhälsan Research Center, Helsinki, Finland; 5 Department of Medicine, Kuopio University Hospital, Kuopio, Finland; 6 School of Educational Sciences and Psychology, University of Eastern Finland, Kuopio, Finland

**Keywords:** Primary health care, Self-rated health, Patient activation measurement, Chronic diseases, Aging population

## Abstract

**Background:**

In the aging population, chronic diseases and multimorbidity are common. Therefore, it is important to engage patients in their self-care. The aim of this study was to analyze the relationship between activity in self-care and self-rated health among primary care patients with chronic diseases.

**Methods:**

The data of the present study were derived from a research project on the Participatory Patient Care Planning in Primary Care (4PHC). A total of 605 patients were recruited in the Siilinjärvi Health Center from those patients who were being monitored due to the treatment of hypertension, ischemic heart disease or diabetes.

We evaluated the level of patient’s activity in self-care with the Patient Activation Measurement (PAM). Self-rated health (SRH) was measured with the 5-item Likert scale. An adjusted hypothesis of linearity across categories of PAM and self-rated health was estimated using analysis of covariance (ANCOVA).

**Results:**

It was found that 76 patients had low activity, 185 had moderate while 336 patients had high activity as measured with PAM. Patients with the highest activity were younger, less depressed, had a lower body mass index and a higher level of physical activity than those with the lower activity. Correspondingly, good SRH was perceived by 29, 45 and 67% of the patients in these three PAM groups adjusted with sex, age, depressive symptoms (BDI) and number of diseases. There was a significant linear trend (adjusted with age, number of diseases and depressive symptoms) between SRH and PAM, *p* < 0.001.

**Conclusions:**

Activity in self-care had an independent, linear relationship with the self-rated health. The present findings suggest that Patient Activation Measurement has the potential to categorize the patients according to their perceived health and their needs related to their disease management and self-care. The present results warrant longitudinal studies on the impact of promoting patient activation levels.

**Trial registration:**

ClinicalTrials.gov Identifier: NCT02992431. Registered 14 December 2016 https://clinicaltrials.gov/ct2/show/NCT02992431

## Background

Populations around the world are rapidly ageing. The World Health Organization has stated that ageing presents both challenges and opportunities; it will increase the demand for primary health care and long-term care, require a larger and better-trained workforce and intensify the need for environments to become more age-friendly [1]. By 2035, every third resident will be over 65 years old in every second municipality in Finland [2].

About 30% of European residents have at least one chronic disease and chronic diseases now affect the vast majority, more than 80%, of people aged over 65 years in Europe. Hence, the numbers of chronic diseases and people suffering from long-term health problems are growing. This development is placing a heavy burden on the healthcare system both with respect to cost and workforce [3].

Patient engagement and patient activation are major focuses of health policy efforts [4–6]. A large proportion of chronic diseases, for example hypertension, ischemic heart disease and diabetes, requires the patient to take active actions daily, called self-care [7–10]. Hence, patients themselves, through their own daily actions and choices, determine to a large extent their need for care and health care outcomes. Therefore, it is essential to devise tools to pinpoint those patients who need most professional help and support in their self-care. We need also more individually tailored processes to support those patients who will be more active in their self-care.

One way to define self-care or self-management is “learning and practicing the skills necessary to carry on an active and emotionally satisfying life in the face of a chronic condition”. Three different types of self-management tasks have been identified: medical (taking medication, adhering to exercise or a diet etc.), role (adapt to maintain, change, or create new meaningful behaviors or life roles) and emotional management (i.e. how to deal with the emotional sequel of having a chronic disease and its consequences to the future life) [7, 10]. There is convincing evidence that patients who are more active in their self-care generally have better health outcomes and a better health-related quality of life [11–15]. In addition, healthcare costs seem to be lower in these individuals [11, 12, 15–17].

The patient activation measurement (PAM) is one widely used tool to study patient activation in self-care [13]. According to Hibbard, patient activation comprises of the degree of knowledge, confidence, and skill that patients have to manage their overall health, and can be seen as a developmental process [13]. Results can be divided into four validated stages of activation. Those at the low end of the PAM scale, are typically passive recipients of care and do not believe in the need for an active patient role, whereas those at the high end of the activation scale, are proactive about their health and engage in many recommended health behaviors [15]. The patient activation theory is rooted in the concepts of self-efficacy, locus of control and in the transtheoretical model of change [18].

Self-rated health (SRH) defined with one question is a valid measure according to population-based studies. SRH has an independent prognostic value regarding mortality, the course of diseases and use of health services [19–22]. Self-rated health may have an independent role in evaluating the disease burden and can be useful in planning and tailoring the care of people with several chronic conditions in primary care [19]. The most important hypothesis to explain the relationship between SRH and all-cause or disease-specific mortality is that subjective assessment of SRH is also a sensitive measure of objective health status [20].

Good SRH has been proven to be directional to favorable health behaviors [23] and it has predicted a better patient self-care activity in the follow-up [24]. However, there is still very little knowledge about the relationship between activity in self-care and SRH, which we aimed to investigate in this present study.

## Methods

The data of the present study were based on the Participatory Patient Care Planning in Primary Care (4PHC, ClinicalTrials.gov Identifier: NCT02992431). This study was conducted in Siilinjärvi municipality which was home to 21,657 residents at the end of the year 2017. In Finland, the primary health care is tax based and distributed to all residents by community health centers provided by the municipalities [25]. The Siilinjärvi Health Center is the main primary health care provider in the Siilinjärvi municipality. In 2017, the mean-age of the population in Siilinjärvi was 41 years. In 2020, the proportion of over 65-years old residents will be 20% [26]. The study population was based on those adult residents (age > 18 years) living in the municipality of Siilinjärvi having diabetes, ischemic heart disease or hypertension.

The participating patients were recruited from those individuals who had a scheduled follow-up visit due to their disease between February 2017 and March 2018. A total of 800 patients who were registered into the electronic patient records in Siilinjärvi Health Center and were being followed-up due to the treatment of hypertension, ischemic heart disease or diabetes were informed about the study. The patients received a study information letter together with a letter inviting them to the follow up visit. The study nurse phoned them and asked about their willingness to participate in the study after 1 week from the letter.

Patients were asked to come for their annual follow-up visit with a nurse and if necessarily, with the general practitioner. Before that follow-up visit, they had received a questionnaire through the post including basic questions about their health, diseases, treatment, and self-care. A total of 605 patients agreed to participate and signed the informed consent. Of these, 597 patients completed all the data needed for this analysis. Each patient’s activity in self-care was measured with the Patient Activation Measurement (PAM). In PAM, patient activation consists of the degree of knowledge, confidence, and skill that patients have to manage their overall health and health care [13]. It is made up of 13 statements about managing one’s health such as “I am confident that I can tell a doctor my concerns, even when he or she does not ask” which respondents answer on a 4-point Likert-type scale (disagree strongly, disagree, agree, agree strongly and N/A) [13]. The responses to items are summed and normalized to a 100-point scale, with higher scores reflecting higher levels of activation. The results can be divided into four validated stages of activation. Patients at PAM Level 1 (score 0–47.0) are Not taking an active role; at Level 2 (47.1–55.1) they are Gaining confidence and knowledge to take action; at Level 3 (55.2–67.0) patients are Taking action and finally at Level 4 (67.1–100) patients are Maintaining behaviors and pressing ahead [13, 14, 27]. In our study, the patients in the PAM level 1 (*n* = 30) and 2 (*n* = 46) were pooled together due to the small number of patients in both groups.

SRH was measured by asking the patients in a question form on how they would rate their health in general. The answer options ranged from excellent (I), very good (II) good (III), fair (IV) to poor (V) [22].

The number of drinks per week and current smoking habits (yes or no and number of cigarettes per day) were asked in question form. The FIT index was used to assess physical activity level and was calculated according to the participants’ responses on an activity questionnaire scale: [FIT = Frequency (F) x Intensity (I) x Time (T)]. Each participant’s frequency was determined by how many days per week he or she exercised on a five-point scale with 1 representing less than once per month and 5 representing 6 or 7 times per week. Intensity was set on a five-point scale with 1 representing light aerobic activity such as normal walking and 5 representing high intensity exercise such as running. Time was determined on a four point scale with 1 representing less than 10 min and 4 referring to greater than 30 min. FIT scores ranged from 1 (low activity) to 100 (high activity) and were divided into four classes 0–12 points, 13–36 points, 37–63 points and over 64 points [28, 29].

Depressive symptoms were measured with the 21-item Beck Depression Index (BDI-21) [30].

The nurse measured the patient’s weight, height, waist, and blood pressure. The plasma concentrations of fasting glucose, hemoglobin A1c (HbA1c) and low-density lipoprotein (LDL) cholesterol were determined according to the standard laboratory protocol of the Kuopio University Hospital district. The descriptive statistics are presented as means with standard deviation (SD), as medians with interquartile range (IQR) or counts with percentages. The unadjusted hypothesis of linearity across categories of PAM levels and Characteristics of the study population were tested using the Cochran–Armitage test or analysis of variance with an appropriate contrast. An adjusted hypothesis of linearity across categories of PAM and self-rated health was estimated using analysis of covariance (ANCOVA). Models included sex, age, BDI and the number of diseases as covariates. The bootstrap method was used when the theoretical distribution of the test statistics was unknown or in the case of violation of the assumptions (e.g. non-normality). Correlation coefficients with 95% CI were calculated by using the Spearman method. The normality of variables was evaluated graphically and by using the Shapiro–Wilk W test. Stata 16.0 (StataCorp LP, College Station, TX, USA) was used for the statistical analyses.

## Results

**Fig. 1:**
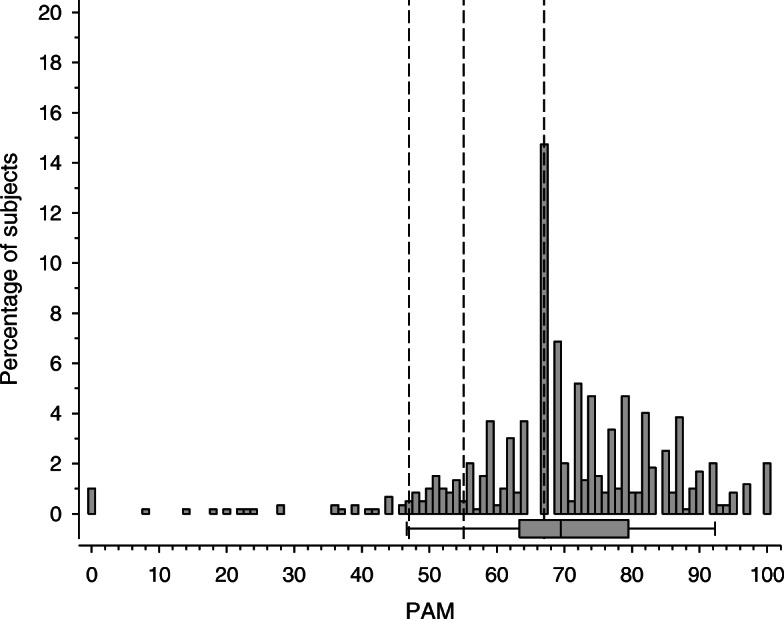
Distribution of the Patient activation measurement (PAM) total score. Box-and-whiskers plot shows median with IQR, and whiskers indicate 5th and 95th percentiles. The dotted lines show the four PAM categories (activation levels): Level 1: Not taking an active role; Level 2: Gaining confidence and knowledge to take action; Level 3: Taking action and Level 4: Maintaining behaviors and pressing ahead

The mean of patient activation score among these typical primary care patients was 69.9 (SD 15.7). The distribution of the PAM score among the patients is shown in Fig. 1.

**Table 1:** Characteristics of the study population of Participatory Patient Care Planning in Primary Care (4PHC) study according to the patient activation measurement (PAM)

	PAM® level			P for linearity
	1–2 *n* = 76 (13%)	3 *n* = 185 (31%)	4 *n* = 336 (56%)	
Women, n (%)	40 (53)	105 (57)	166 (49)	0.41
Age, mean (SD)	72 (10)	70 (8)	68 (9)	< 0.001
Living with a spouse, n (%)	47 (62)	122 (66)	240 (71)	0.075
Number of education years, mean (SD)	9.7 (3.3)	9.8 (2.9)	10.4 (3.2)	0.021
Retired, n (%)	67 (88)	165 (89)	279 (83)	0.14
Smoking, n (%)	9 (12)	20 (11)	36 (11)	0.79
Number of alcohol drinks per week, mean (SD)	2.1 (5.4)	1.7 (3.0)	1.9 (3.8)	0.75
Physical activity^a^, mean (SD)	29 (20)	37 (18)	45 (20)	< 0.001
Body mass index, kg/m2, mean (SD)	31.7 (7.2)	29.8 (5.9)	28.8 (5.2)	< 0.001
Blood pressure, mmHg, mean (SD)				
Systolic	144 (17)	147 (19)	145 (18)	0.99
Diastolic	80 (10)	82 (11)	82 (10)	0.16
Fasting plasma glucose, mmol/l, mean (SD)	6.79 (1.59)	6.70 (1.26)	6.48 (1.25)	0.065
HBA1C, mmol/mol, mean (SD)	42.7 (11.0)	42.1 (9.3)	40.5 (7.2)	0.057
LDL, mmol/l, mean (SD)	2.58 (1.05)	2.68 (1.03)	2.65 (0.93)	0.70
Beck depression index score, mean (SD)	9.2 (7.0)	6.4 (5.0)	5.2 (4.6)	< 0.001
Diseases, n (%)				
Diabetes mellitus	35 (46)	84 (45)	127 (38)	0.11
Hypertension	63 (83)	155 (84)	267 (79)	0.37
Ischemic heart disease	20 (26)	50 (27)	97 (29)	0.61
Atrial fibrillation	14 (18)	23 (12)	37 (11)	0.083
Cardiac failure, insufficiency	5 (7)	16 (9)	18 (5)	0.50
Musculoskeletal disorders	51 (67)	102 (55)	168 (50)	0.006
Dementia	5 (7)	2 (1)	0 (0)	< 0.001
Depression or other psychiatric disorders	6 (8)	12 (6)	18 (5)	0.38
Asthma or chronic obstructive pulmonary disease	14 (18)	20 (11)	49 (15)	0.58
Neurological diseases	1 (1)	0 (0)	7 (2)	0.36
Cancer	2 (3)	2 (1)	4 (1)	0.37
Number of diseases, mean (SD)	2.8 (1.4)	2.5 (1.2)	2.4 (1.2)	0.004

^a^Kasari FIT index

It was observed that 76 patients had a low activity (levels 1 + 2), 185 had moderate and the majority, 336 patients, high activity as measured with PAM. The age was significantly associated with the PAM level the most active group being younger than the two other groups. In the higher activity group, patients had more educational years. Body mass index was lower in the higher activity groups. In the highest activity group, patients had a higher level of physical activity meaning that they exercised more, and they were less likely to suffer from musculoskeletal diseases. Otherwise there were no differences in the number of diseases between the three groups. There were no statistically significant differences in blood pressure, plasma glucose, HbA1C or LDL-cholesterol (Table 1).

**Fig. 2:**
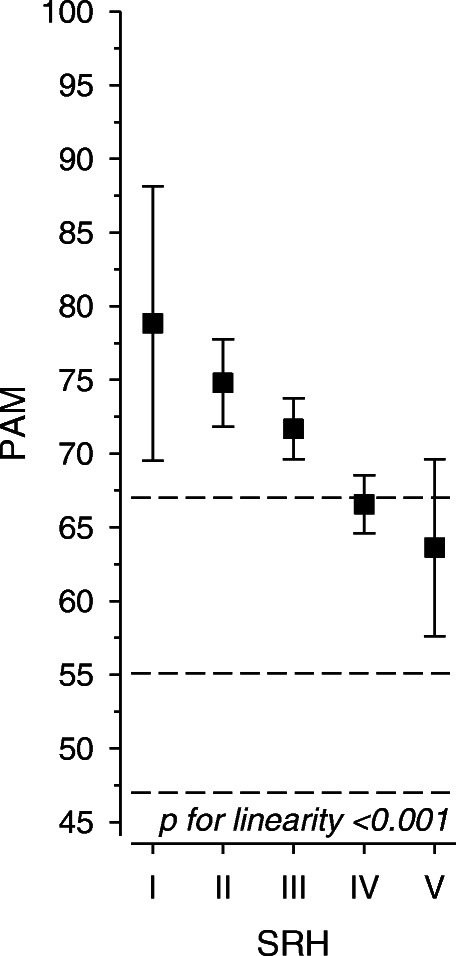
Patient activation measurement (PAM) score according to self-rated health (SRH), adjusted by sex, age, BDI (Beck depression index) index and the number of diseases. Whiskers represent 95% confidence intervals. SRH category I means excellent health and V poor health, respectively. Hatched lines represent the four PAM categories

When adjusted with sex, age, BDI-score and the number of diseases, there was a significant linear trend between SRH and PAM, *p* < 0.001 (Fig. 2). The patients who felt themselves healthiest had the highest activity. Correspondingly correlation between SRH and PAM score was 0.34 (95% CI: 0.37 to 0.41).

## Discussion

The present study examined the relationship between self-rated health (SRH) and patient activation measurement (PAM) in a primary care setting in those patients suffering from common chronic diseases. Patient activation had a significant independent linear relationship with self-rated health. As far as we are aware, this is the first study focusing on this particular issue in primary health care among patients with diabetes, ischemic heart disease or hypertension.

The SRH represents a valid patient related outcome i.e. the reliable assessment of disease burden, risk of mortality and use of health services [19–22]. In addition, the SRH has an ability to provide relevant information about the cardiovascular risk in addition and irrespective of traditional risk factors [31]. Patient activation as measured with PAM is related to the treatment adherence, lifestyle factors, health related outcomes and cost [11–17]. PAM has been validated also in Finland [32]. Previously, PAM and SRH have been measured from the same patients in PAM validation studies in Europe and USA [33–36]. The general results emerging from these studies have indicated that PAM could be higher in people with better SRH, but they did not analyze this relation further and consider other background factors potentially influencing that relationship.

In the present study population of patients having a chronic disease, the subjects with high PAM-score usually perceived their health as good in contrast to those having lower scores. Furthermore, in accordance with previous studies, the subjects with lower activity were more obese [15], had lower physical activity [37] and more depressive symptoms [38] and more often musculoskeletal disorders [39] and multimorbidity [40]. However, there were no significant differences in other risk factors (blood pressure levels, LDL cholesterol, smoking and alcohol use) between the PAM categories. In accordance with our findings, a previous study found that patients with higher activation did not have more often normal levels of LDL, diastolic blood pressure and HbA1c than the patients with lower activation [15]. Patients’ age was inversely related to the PAM category. In general, older people scored lower on the PAM scale than their younger counterparts. For example, in a population-based study from the U.K., 72% of the respondents with at least one chronic condition (aged at least 65 years, mean age 75 years) had PAM level 1 or 2 at the baseline of the study [40], the corresponding proportion being 13% in the present study.

In addition to increasing numbers of chronic diseases, the relationship between age and PAM score might reflect the duration of the diseases, because often a deterioration of health occurs in individuals with a chronic illness with time [24]. Over the course of time, patients may experience multiple failures in trying to manage chronic illness and that could lead to discouragement and disempowerment and finally make patients passive about their health [41]. Furthermore, the older multimorbid patients may be more likely than younger people to delegate authority to health workers [42].

Regardless of age, multimorbidity and depressive symptoms, SRH categories had a significant and linear relationship with the PAM score adjusted with age, number of diseases and depressive symptoms. Furthermore, the present findings suggest that higher levels of PAM indicate active health behavior and positive patient reported outcomes reflecting good self-rated health. From the health benefit point of view, PAM seems to be both reasonable and feasible. For example, every additional 10 points in PAM in a study concerning primary care patients in the United States predicted a 1% smaller probability of having an emergency department visit, being obese or smoking. The likelihood of having clinical indicators in the normal range was 1% higher in the same study [15].

The mean PAM in our study was 69.9 points. In a previous Finnish study conducted in primary care patients (*N* = 137) with diabetes, hypertension and hypercholesterolemia, the mean PAM was 63.6 [32]. In that study, the patients were about 10 years younger than those examined in our study. In the PAM validation studies emerging from Europe and USA, the mean PAM varied between 61.2–67.1 points being lowest in Netherlands and highest in Germany [33–36]. The study from the Netherlands represented members of the Dutch National Panel of people with Chronic illness or Disability. In the German study, the patients were the members of the Health fund who had a minimum of one chronic disease.

In the present study, over half of the patients were at the highest activation level 4, about one out of every three were at level 3, with the rest being in levels 1 and 2. These percentages have been somewhat different from the former studies varying in level 4: 14–56%, level 3: 23–38%, level 2: 9–37% and level 1: 6.8–18.5% [12, 14, 24, 43]. In our study, we have used the original limit for the level four (above 67.1 points) whereas in some more recent studies, the limit seems to be somewhat higher i.e. 72.5 points [16, 44]. Thus, when compared to previous studies, this might have changed the group size at levels 3 and 4 to some extent. However, the point limits were not reported on all of the articles mentioned above.

The time after setting the diagnosis of a chronic disease also seems to exert an influence on the PAM i.e. being diagnosed with a severe condition can exert a positive change in a patient’s level of activation [32]. However, accepting that one has a chronic illness is a demanding process. A Patient Health Engagement (PHE) model including four evolving phases (blackout, arousal, adhesion and eudaimonic project) is one way to explain the process. This highlights how patients emotionally elaborate and make sense of their illness status and identity as a patient, and it may be considered as a crucial precursor of their ability and willingness to play a more active role in self-management [18]. From the patient’s perspective, good health means feeling well, not necessarily simply a good clinical outcome or being active [45].

The real-life primary care setting is one strength of the present study. The participation rate was high, almost 80 % of informed patients agreed to participate. The data consisted of both self-assessments and clinical data. The present study is truly representative of the primary care patients in a Finnish Health Care center. All the studied diseases are common in the Finnish population and thus patients need to cope with them on a day-to-day basis. The age of the patients is quite typical for patients in Finnish Health Centers.

One weakness of the study is that it examines patients from only one area in Finland which can impact on the generalization of the results in Finland or globally. We can also consider as a weakness the fact that we were unaware of the duration of the illnesses.

In our study, the patients were interested in their health because they had visited the health care center already before and they agreed to participate the follow-up of their chronic disease in primary care. There were patients with several chronic conditions and in that respect, they represented well the Finnish primary care patients. In our study, more than half of the patients with diabetes, hypertension or ischemic heart disease were in PAM level 4 indicating that these patients were proactive about health and were engaging in many recommended health behaviors [13]. These patients in PAM level 4 perceived their health most often as good. Furthermore, those patients were the least obese, were most often physically active and had the least depressive symptoms. However, we do not have information about the disease management and how the follow-up had been tailored and resources allocated. PAM could be a potential tool for these purposes also in the Finnish primary care.

## Conclusions

The Patient Activation Measurement had a linear relationship with the self-rated health when potential confounders including sex, age, the number of diseases and depressive symptoms were considered. The present findings suggest that it has a potential in categorizing patients according to their perceived health, and their needs related to disease management and self-care, which can help in providing more tailored care to the patients and allocate resources more efficiently. However, based on a cross-sectional study, recommendation about the use of PAM to increase patients’ self-care activities in order to improve disease management cannot be given. To further study the impact of PAM on disease management and health outcomes as well as to understand and take advantage of this new knowledge in practice, we need to explore the relationship between patient activation with the disease specific and relevant patient reported outcomes in a longitudinal setting.

## Data Availability

The data of the current study are not publicly available due to protection of individual privacy but are available from the corresponding author on reasonable request.

## Abbreviations

PAM: Patient activation measurement
SRH: Self-rated health
